# Enhanced emission of charged-exciton polaritons from colloidal quantum dots on a SiN/SiO_2_ slab waveguide

**DOI:** 10.1038/srep09760

**Published:** 2015-05-19

**Authors:** Xingsheng Xu, Xingyun Li

**Affiliations:** 1 State Key Laboratory of Integration Optoelectronics, Institute of Semiconductors, Chinese Academy of Sciences, Beijing 100083, China

## Abstract

We investigate the photoluminescence (PL) spectra and the time-resolved PL decay process from colloidal quantum dots on SiN/SiO_2_ wet etched via BOE (HF:NH_4_F:H_2_O). The spectrum displays multi-peak shapes that vary with irradiation time. The evolution of the spectral peaks with irradiation time and collection angle demonstrates that the strong coupling of the charged-exciton emission to the leaky modes of the SiN/SiO_2_ slab waveguide predominantly produces short-wavelength spectral peaks, resulting in multi-peak spectra. We conclude that BOE etching enhances the charged-exciton emission efficiency and its contribution to the total emission compared with the unetched case. BOE etching smoothes the electron confinement potential, thus decreasing the Auger recombination rate. Therefore, the charged-exciton emission efficiency is high, and the charged-exciton-polariton emission can be further enhanced through strong coupling to the leaky mode of the slab waveguide.

Photoluminescence (PL) intermittence, or blinking, is the greatest obstacle to the application of colloidal quantum dots (CQDs). The generally accepted explanation is that the dark state in blinking is caused by photo-charging in quantum dots^1^. Under photo-excitation, electrons are ejected from the dot to outer defect states, thus forming positively charged quantum dot. Residual electron-hole pairs decay rapidly to the ground state through non-radiative Auger relaxation, quenching any emission^2^. There is some probability for the CQD core to be neutralized, thus making the CQDs bright again^3^. If the nanocrystals are charged, the extra carrier triggers non-radiative Auger recombination between the extra charge and a subsequently excited electron-hole pair. In early studies, the rate of Auger recombination was typically orders of magnitude faster than the rate of radiative recombination^4^, which suppressed the PL almost completely in charged nanocrystals^5^. PL would resume only after the CQD was neutralized again.

With more recent developments in synthesis technology, it has become possible to produce CQDs in which the charged-exciton-induced PL quenching is not complete; instead, the charged-exciton emission merely modifies the shape of the PL spectrum. In a study of the PL spectra of type-II CdTe/CdSe core/shell CQDs, the charged-exciton (trion) emission induced a blue shift in the spectrum^6^. X. Y. Wang *et al.* have synthesized CdZnSe/ZnSe quantum dots and found that these CQDs produce strong PL despite being charged. The corresponding lifetime was short, and the PL contained multi-peaks. These authors utilized a radially graded alloy of CdZnSe into ZnSe to soften the abrupt confinement potential of the CQDs, which helps explain the unusual PL properties of the CdZnSe/ZnSe CQDs^7^. In a previous study, colloidal CdSe/CdS CQDs were synthesized, and it was found that the negatively charged exciton PL quantum yield reached 100% at low temperatures, where the Auger recombination was completely suppressed^8^. The abrupt boundary represented by the CdS outer surface and the temperature-dependent delocalization of one of the trion electrons from the CdSe core enhanced the Auger recombination in these CQDs. Thermal delocalization of one of the electrons from the CdSe core into the CQD shell induced Auger recombination, and the delocalized electron then interacted with the abrupt outer surface of the CQD to stimulate non-radiative Auger recombination. As the temperature was decreased to below 200 K, the CQDs became permanently negatively charged. However, when the temperature was decreased to 30 K, the CQD quantum yield increased to 100%, and the PL lifetime decreased with decreasing temperature^8^.

The charging of CQD may be related to its surfaces and its environment. The presence of acceptors, such as TiO_2_^9^, indium tin oxide (ITO)^10^, inorganic complexes^11^, C60^12^, and polymers^13^, which will accept an electron transferred from a photo-activated CQD, will result in charge-separated states. It has been observed that the PL quantum efficiency of CdSe CQDs decreased when an electron was accepted from p-phenylenediamine. However, the efficiency could be increased by the suppression of non-radiative recombination resulting from the passivation of surface defects in CdSe CQDs with n-butylamine^14^,^15^. Photo-charging leaded to bleaching of the steady-state absorption and partial quenching of the emission^16^.

When CQDs are embedded in an optical cavity or waveguide, the emission from the CQDs will couple to cavity or waveguide. In the strong-coupling regime, an exciton and a photon can interact with each other, and they can exchange energy on a time scale shorter than their lifetimes. The exciton and photon will then form a hybrid state called exciton polariton. An exciton polariton can be regarded as a quasi particle with mixed properties of light and matter^17^. This state has important applications in fields involving light matter interactions, such as in Bose-Einstein condensation (BEC)^18^, low-threshold coherent emission^19^, and low-threshold all-optical switches^17^,^20^. To date, waveguide exciton-polariton emission from colloidal quantum dots has been rarely reported.

In this report, by using a very simple wet etching method, the coupling between the charged-exciton emission from CQDs and the leaky mode of the SiN/SiO_2_ film on which the CQDs were deposited is investigated. From the collection-angle-dependent PL spectra, it was determined that the emission of the CQDs strongly coupled to the leaky modes of the SiN/SiO_2_ slab waveguide. As evidenced via the measured wavelength-dependent lifetime, the short-wavelength peaks in the multi-peak spectra predominantly originated from charged-exciton emission coupled to the leaky modes, leading to the formation of waveguide charged-exciton polaritons. In our system, the weight of the charged-exciton emission component compared with the total PL was much higher than those observed from conventional material without BOE etching and CQDs on silicon dioxide. For the system subjected to BOE wet etching, the lifetime of the charged-exciton emission was increased and the efficiency of the charged-exciton emission was higher than the corresponding values for CQDs on SiN without BOE wet etching. BOE-etched SiN/SiO_2_ may have two important effects on the optical properties of the CQDs: 1) the BOE etching changes the surface Fermi level of the SiN, facilitating the transfer of electrons from the CQDs to the SiN and enhancing the charging degree of the CQDs, and 2) light irradiation of the BOE-etched SiN/SiO_2_ will increase the Coulomb repulsion, which may soften the electron confinement potential of the CQDs. The enhanced charging of the CQDs and the softened electron confinement potential can enhance the lifetime and PL efficiency of the charged-exciton emission. This means that BOE-etched SiN/SiO_2_ will suppress the Auger recombination in CQDs. After these modifications, the leaky modes of the SiN/SiO_2_ will enhance the charged-exciton emission at the corresponding wavelength and the strong coupling between the charged-exciton emission and the leaky modes, leading to the formation of charged-exciton polaritons. Therefore, we have developed a very simple method and structure for the suppression of the non-radiative Auger recombination rate and the enhancement of the charged-exciton emission, and our research will has potential applications in high-efficiency optoelectronics.

## Results

### PL spectra of CQDs on a BOE-etched SiN/SiO_2_ film with light irradiation time

**Figure 1:**
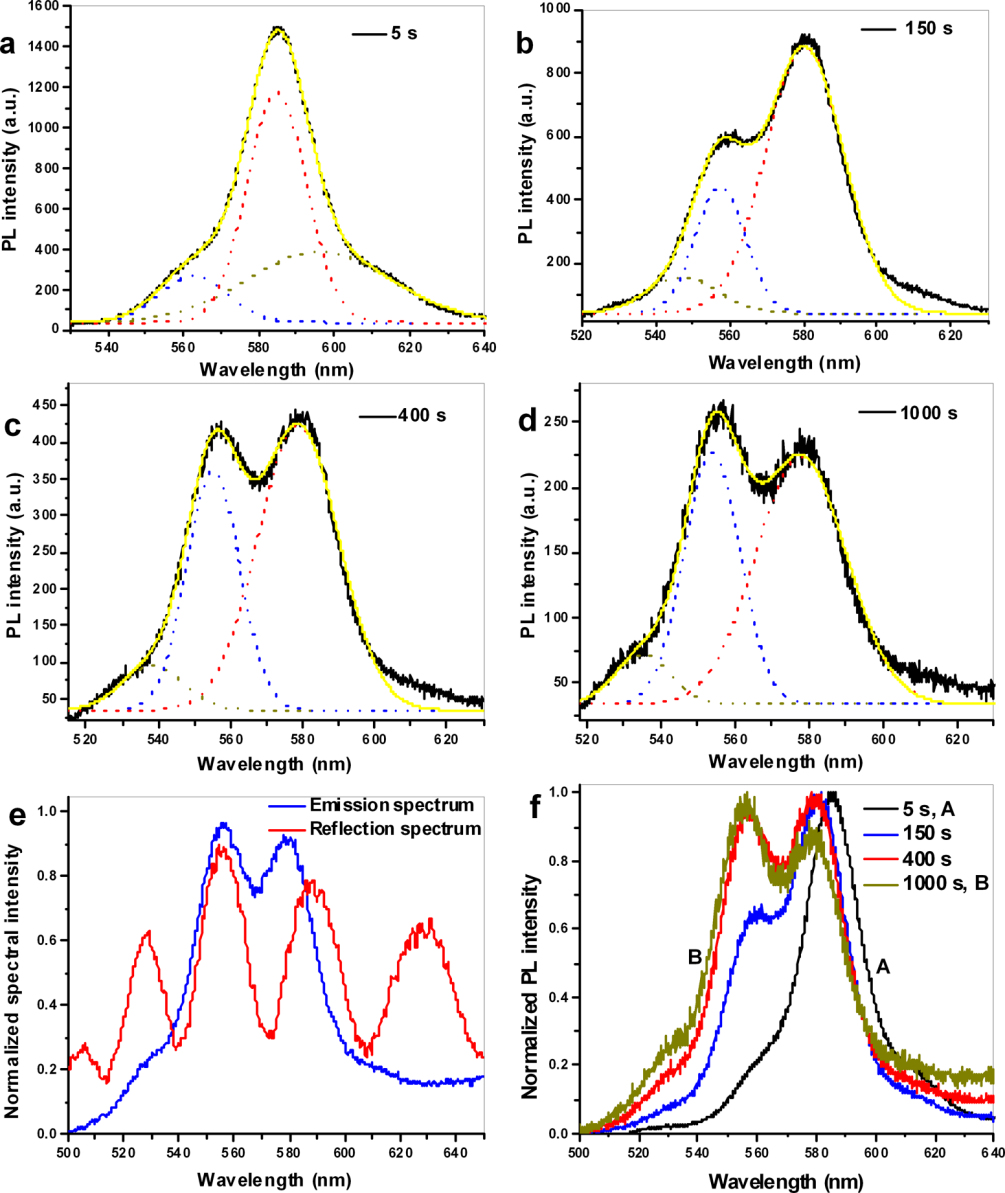
The PL spectra of CQDs on a BOE-etched SiN/SiO_2_ film for various irradiation time: (a) 5 s, (b) 150 s, (c) 400 s, (d) 1000 s. (a)–(d) The yellow curved lines represent three-term Gaussian fits, the dotted-curves are the Gaussian fits. (e) The emission spectrum and the reflection spectrum of CQDs on BOE-etched SiN of 420 nm in thickness. (f) Comparison of spectra from 5 s to 1000 s.

CQDs on BOE-etched SiN/SiO_2_ film was excited by a 405 nm continuous-wave laser diode with a 40× objective, and the emission spectrum was collected using the same objective. The samples were excited under an excitation power of 2.0 mW for different irradiation time and the spectra were collected automatically using a spectrometer with a Si CCD at an interval of one frame per second. The measured spectra are presented in Fig. 1,Fig. S1 and Fig. S2. The spectrum acquired after 5 s is presented in Fig. 1a to represent the initial state of the material; it is centered at 585.4 nm, and there are two weak spectral peaks, one on the long-wavelength side and one on the short-wavelength side of the main peak. As the irradiation time increased to 100 s, the total PL intensity increased slightly because of photo-oxidation^21^, and the centre wavelength shifted to 583.7 nm (Fig. S1a). The PL intensity of the short-wavelength side peak also slightly increased compared with the spectrum presented in Fig. 1a. With a further increased irradiation time of 150 s, the main peak blue shifted to 580.9 nm, and the intensity of the short-wavelength side peak increased to more than half the intensity of the main peak, whereas the long-wavelength side peak nearly disappeared (Fig. 1b). As the irradiation time increased to 400 s, as shown in Fig. 1c, the intensity of the short-wavelength side peak increased to become comparable to that of the main peak. The spectrum acquired after an irradiation time of 1000 s is presented in Fig. 1d; here, the intensity of the short-wavelength peak is greater than that of the original ‘main’ peak at 580.2 nm.

The CQD emission spectrum and the reflection spectrum from the slab (representing the leaky modes of the slab waveguide) are compared in Fig. 1e. The blue line represents the emission spectrum from the CQDs at an irradiation time of 1000 s, and the red line represents the reflection spectrum from the SiN/SiO_2_ film. The emission peak at the short-wavelength side of the spectrum nearly overlaps with a peak of the reflection spectrum, which corresponds to CQD emission coupled to the waveguide leaky mode. The long-wavelength peak in the emission spectrum is located near another reflection peak, but its wavelength is shorter than that of the reflection peak. These peaks in the emission spectrum originated from CQD emission coupled to the SiN waveguide leaky mode. The deviation between the emission peak and the reflection peak originated from the spectral blue shift caused by photo-oxidation.

The normalized spectra from irradiation time of 5 s to 1000 s are compared in Fig. 1f. The spectrum on the right, *A*, was acquired after a short irradiation time of 5 s, and the spectrum on the left, *B*, was acquired after an irradiation time of 1000 s. The initial spectrum from the CQDs, represented by spectrum *A*, was fitted to tri-Gaussian function (Fig. 1a). The center wavelength, spectral width and contribution to the total emission of the short-wavelength component were found to be 562.61 nm, 17.99 nm and 0.12, respectively. The corresponding values for the middle-wavelength component are 584.78 nm, 15.78 nm and 0.51, respectively, and those for the long-wavelength component are 596.05 nm, 37.96 nm and 0.37, respectively. Thus, in spectrum *A*, which is centered at 584.78 nm, there are two side peaks located at 562.61 nm and 596.05 nm. When spectrum *B*, corresponding to the long irradiation time, was fitted to a tri-Gaussian function (Fig. 1d), the center wavelengths of the three components were found to be 535.07 nm, 553.78 nm, 577.85 nm, respectively. The corresponding spectral widths are 14.87 nm, 15.14 nm, and 24.37 nm, respectively, and the contributions to the total emission of the three components are 0.07, 0.36 and 0.57, respectively. In Fig. 1f, the center peak of spectrum *A* at 584.78 nm represents the initial emission from CQDs, whereas the two side peaks at 562.61 nm and 596.05 nm are attributable to the coupling of the CQDs emission to the slab waveguide leaky modes. The 577.85 nm peak of spectrum *B* represents the original emission from the CQDs and its coupling to a slab waveguide leaky mode, whereas the peaks at 535.07 nm and 553.78 nm represent the coupling of the CQD's emission to slab waveguide leaky mode. The PL intensity of the 553.78 nm peak in spectrum *B* (long irradiation time) is much stronger than that of the peak of 562.61 nm in spectrum *A* (short irradiation time). This finding indicates that the coupling of the emission to the waveguide leaky mode was enhanced with increasing irradiation time before the peak wavelength of the spectra overlap with the slab waveguide leaky mode. Upon photo-oxidation, the spectrum blue shifted, and when the spectrum overlapped with the slab-waveguide leaky mode, the multi-peak in the spectrum emerged. From the changes observed in the short-wavelength peak in Fig. 1f, we conclude that this peak originates not only from exciton coupling to the leaky mode but also from the coupling of charged-exciton emission to the leaky mode. Under light irradiation, the CQDs become ionized, and the emission of charged excitons increases. With increasing irradiation time, the positive charged-exciton emission shifts to blue.

Upon BOE etching, the etched-off SiN is very thin (less than 50 nm); both a BOE-etched SiN film and an unetched SiN film can support leaky modes in the emission region of the CQDs (approximately from 520 nm to 620 nm). However, the spectra of CQDs on unetched SiN were found to exhibit very few multi-peak characteristics. The spectrum of CQDs on unetched SiN film is presented in Fig. S1f. This spectrum contains only one peak, which is attributable to the exciton emission from the CQDs. There is no spectral split such as those observed in Figs. 1a–d. This observation may further demonstrate that the short-wavelength peak observed in the previous spectra originated not only from the coupling between the CQD emission and the slab-waveguide leaky modes but also from the charged-exciton emission. The blue shift of the wavelength was related to the charged-exciton emission and the coupling of the charged-exciton emission to the waveguide leaky modes. This judgment was verified by the time-resolved spectra of the CQDs, discussed below.

**Figure 2:**
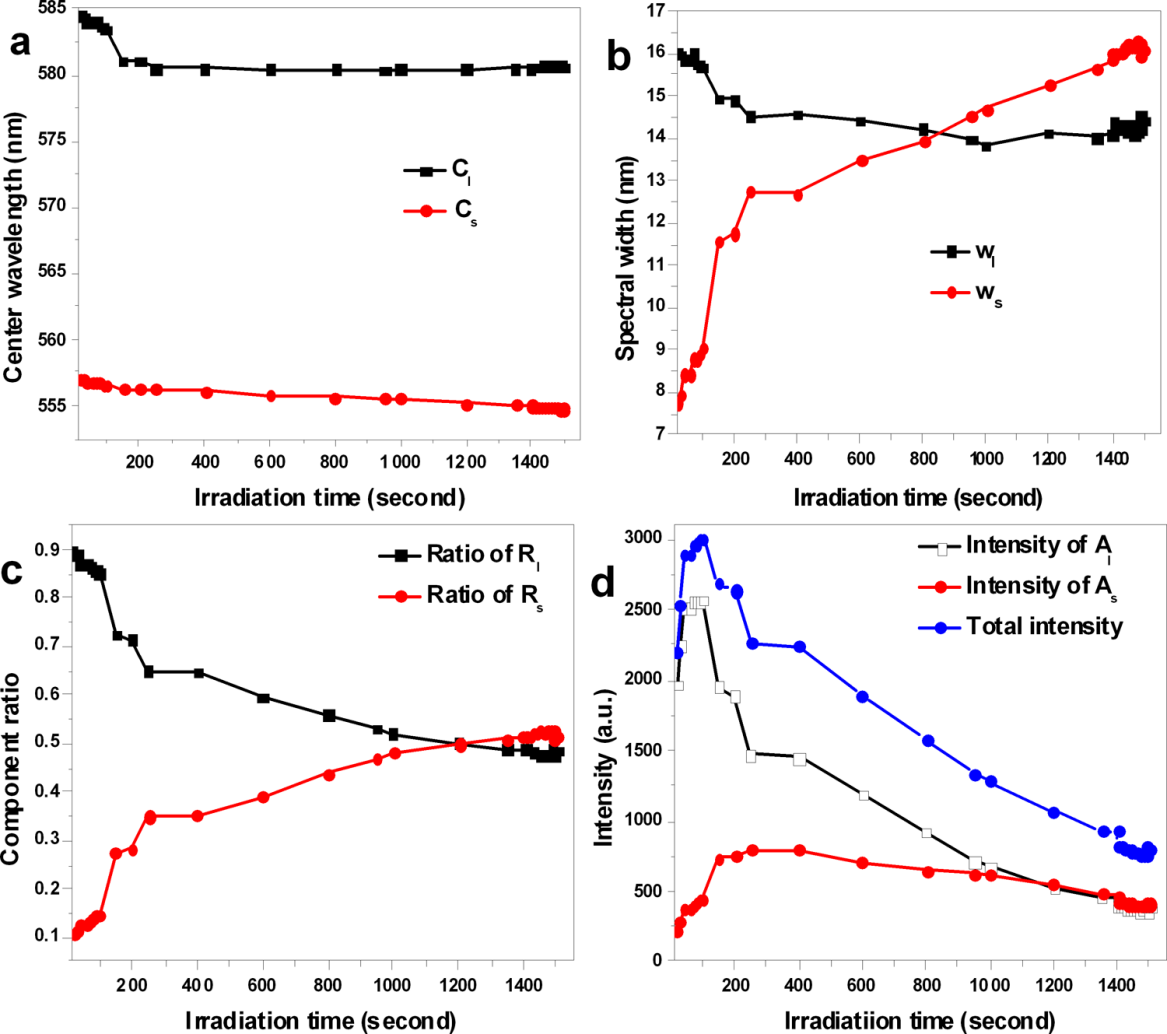
The bi-Gaussian fitted parameters of the spectra presented in Figure 1. The fitted parameters as function of light irradiation time: (a) The center wavelengths. (b) Spectral widths. (c) Ratios of the short-wavelength component and the long-wavelength component to the total emission. (d) PL intensities. In the figure, C_l_, C_s_ are the center wavelengths, W_l_, W_s_ are the spectral widths, A_l_, A_s_ are the PL intensities, of the long-wavelength component and the short-wavelength component, respectively; and R_l_, R_s_ are the ratios of the long-wavelength component and the short-wavelength component to the total emission, respectively.

The spectra acquired at different irradiation time were fitted using a bi-Gaussian function, and the fit parameters are presented in Fig. 2 and Fig. S3. After approximately 1500 s (25 min), the center wavelength of the short-wavelength component in the initial spectrum had shifted from 556.5 nm to 554.8 nm, and the initial center wavelength of the long-wavelength component had shifted from 584.5 nm to 580.6 nm. Both peaks blue shifted slowly as a function of irradiation time (Fig. 2a). As shown in Fig. 2b, the spectral width of the long-wavelength component varied from 16.1 to 14.4 nm, whereas the spectral width of the short-wavelength component dramatically increased from 7.8 to 16.1 nm. The ratio of the long-wavelength component to the total emission decreased from 0.895 to 0.485, whereas the ratio of the short-wavelength component to the total emission increased from 0.104 to 0.514 after 1500 s (Fig. 2c). During the initial stage (0–40 s), the total PL intensity increased dramatically with increasing irradiation time. The intensity of the long-wavelength component also increased dramatically, whereas the intensity of the short-wavelength component increased slowly (Fig. 2d). In the time range 90–250 s, the intensity of the long-wavelength component decreased dramatically, whereas the intensity of the short-wavelength component increased slowly, and the total PL intensity also dramatically decreased (Fig. 2d). As the irradiation time increased, the spectral width and the contribution to the total emission of the short-wavelength component increased, whereas the spectral width and the contribution to the total emission of the long-wavelength component decreased. Initially, the short-wavelength component primarily represented charged-exciton emission, whereas the long-wavelength component primarily represented exciton emission^22^. With increasing irradiation time, the CQD spectrum blue shifted because of photo-oxidation^21^. The long-wavelength component became to convert into the short-wavelength component after the critical time of 250 s; the exciton emission became coupled to the short-wavelength leaky mode of SiN/SiO_2_ slab, and the exciton emission converted into charged-exciton emission. During this process, the spectral width and the contribution to the total emission of the short-wavelength component gradually increased because of the increasing contribution from the charged-exciton emission. At the same time, the spectral width and the contribution to the total emission of the long-wavelength component gradually decreased because of the decrease in the exciton emission. At approximately 850 s, the spectral width of the short-wavelength mode exceeds that of the long-wavelength mode (Fig. 2b), as does its contribution to the total emission at approximately 1200 s (Fig. 2c). Thus, the long-wavelength mode and the short-wavelength mode interact with each other^23^, and the exciton emission and the charged-exciton emission interact with each other. Over time, the energy in the long-wavelength component converted to the short-wavelength component. Fig. S3e displays the data presented in Fig. 2a, which characterizes the modes as a function of the irradiation time, in units of meV instead of nm. At the initial irradiation time of 15 s, the energy interval between the long-wavelength mode and short-wavelength mode was 105 meV. As the irradiation time increased to 250 s, this interval decreased to 93.7 meV. At the critical time of 250 s, the minimum wavelength interval between the two modes is observed. After 1500 s, the interval increased once again to 100 meV. An anti-crossing occurred between the long-wavelength mode and the short-wavelength mode. Therefore, the short-wavelength mode and the long-wavelength mode are strongly coupled to each other, and the excitons and the charged excitons interact with each other.

### Spectra under various collection angles

**Figure 3:**
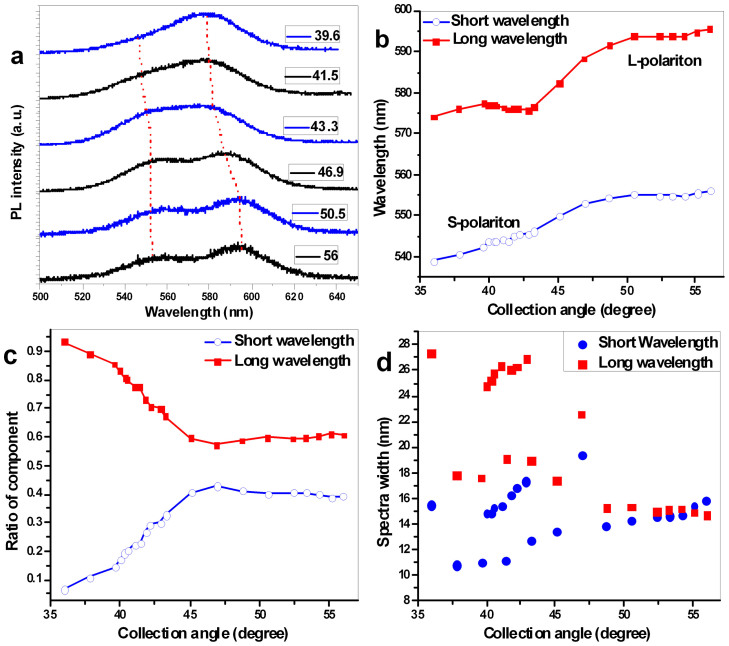
The spectra and spectral parameters as a function of collection angle from 36° to 56°. (a) Representative spectral changes with variations in collection angle. The symbols of the number values represent the collection angles. (b) Center wavelengths for two spectral peaks as the collection angle varies from small to large. (c) Ratios of the long-wavelength and the short-wavelength components to the total emission. (d) Spectral widths as a function of collection angle.

The spectra of the CQDs were measured as a function of collection angles, and some of the representative spectra are presented in Fig. 3a, where the dotted curves indicate the peak-wavelengths trajectory. The center peak wavelength as a function of the collection angle, from small to large (36–56°), are presented in Fig. 3b. The spectrum measured at a collection angle of 36° could be fitted to a Gaussian function. When the collection angle was in the range of 36–43°, the spectrum split, but the interval between the center wavelength of the long-wavelength component and that of the short-wavelength component remained similar. As the collection angle was increased, the center wavelengths of the two components separated from each other. Therefore, the trajectory of these two center wavelengths changed with the collection angle and exhibited anti-crossing phenomenon, which is a sign of a strong coupling effect. As shown in Fig. 3b, the anti-crossing point occurred at a collection angle 43° when the wavelength interval between the two modes was 39 nm and the Rabi splitting was 82 meV. In Fig. 3a and b, the spectral peaks are related to the emission of exciton polaritons; at long wavelength, exciton polaritons are defined as L-polaritons, whereas those at short wavelength are defined as S-polaritons. The evolution of the contributions to the total emission of the two types of polaritons as a function of collection angle is displayed in Fig. 3c. As the collection angle increased from 36° to 47°, the relative contribution of L-polariton decreased from 0.93 to 0.57, whereas that of S-polariton increased from 0.07 to 0.43. As the collection angle was larger than 47°, the contributions diverged in the opposite direction. The evolution of the spectral widths is depicted in Fig. 3d. Near the anti-crossing point, the spectral width exhibited some randomness, which may have arisen because of the strong coupling effect. At points far from the anti-crossing point, especially at angles larger than 47°, the spectral widths of the S-polaritons and L-polaritons were similar. When the angles was smaller than 47°, the coupling of the exciton emission and charged-exciton emission to slab waveguide mode was relatively weak, and the spectral width of exciton emission spectrum was broader than that of the charged-exciton emission as shown in Fig. 3(d). However, as the collection angle reached to 47°, the coupling became strong, both the emission of exciton and that of charged exciton coupled to the slab waveguide, which had significantly effect on the CQDs' exciton emission and charged-exciton emission. Therefore, as the collection angle was larger than 47°, the spectral width of exciton emission and that of charged-exciton emission were similar.

### PL decays at various wavelengths and the fit parameters

**Figure 4:**
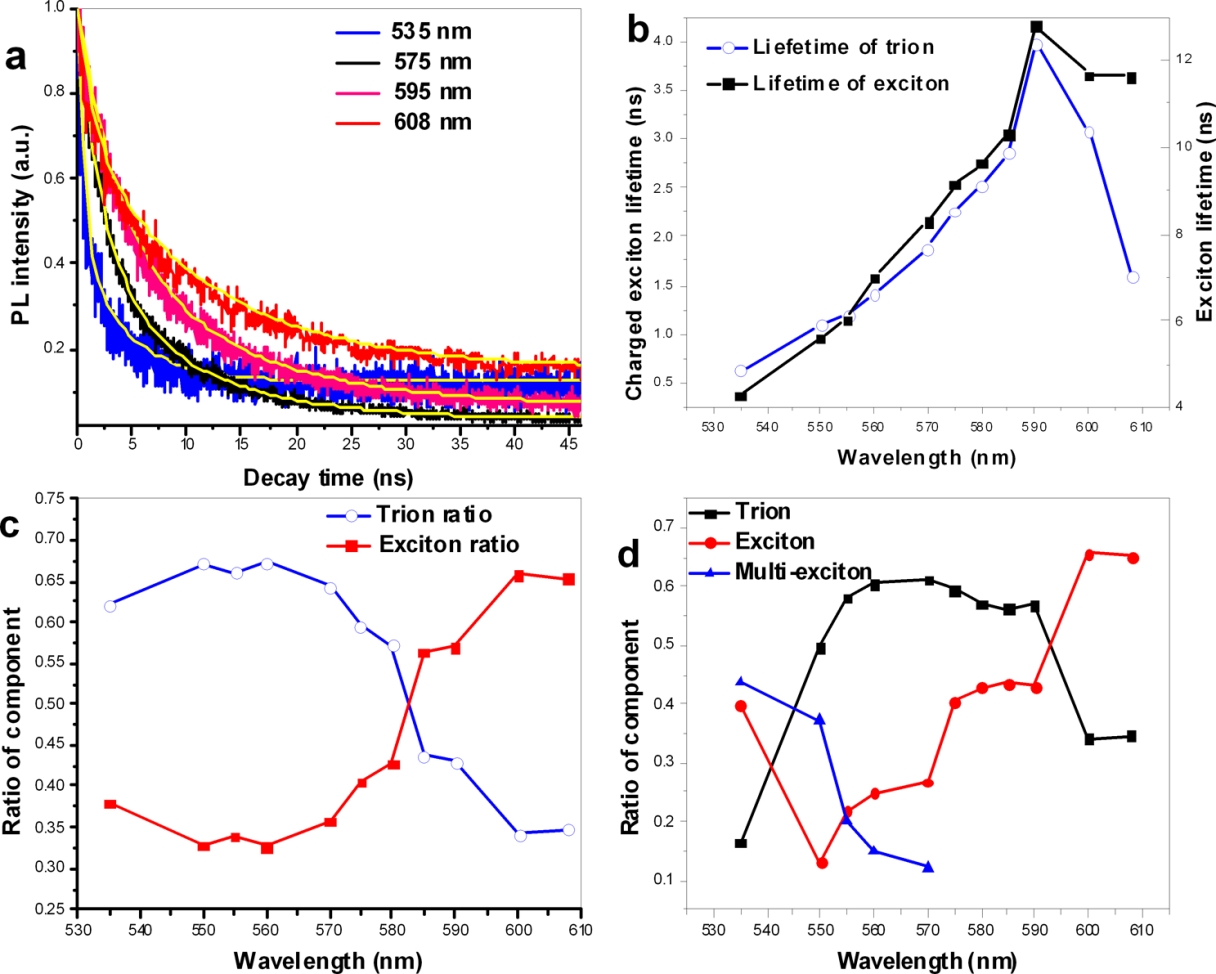
PL decays at various wavelengths and the fit parameters for the PL decays fitted to multi-exponential functions. (a) Comparison of PL decays at various wavelengths: 535 nm, 575 nm, 595 nm, and 608 nm. The yellow lines are fitted curves obtained using a two-term exponential function, and the other color lines represent experimental results. (b) Comparison of the lifetimes for charged-exciton emission and exciton emission. (c) The component contributions from the two-term exponential fitting of PL decays at various wavelengths, (d) The component ratios at various wavelengths from fitting of three-term and two-term exponential functions.

The time-resolved spectra were measured for the same sample used in Fig. 1 under excitation by a pulsed laser with a wavelength of 400 nm and a pulse width of approximately 65 ps. The collected emission from the CQDs was sent to a monochromator with a fast photomultiplier tube connected to a time correlated single photon counting system. The PL decay of the CQDs on BOE-etched SiN/SiO_2_ was measured at various wavelengths, and representative decay curves and their fits using multi-exponential functions are presented in Fig. 4a, Fig. S4 and Fig. S5. When the PL decay was fitted to a two-term exponential, the component with the shorter lifetime was assigned to charged-exciton emission and the component with the longer lifetime was assigned to exciton emission^8^. From the fitted parameters presented in Fig. 4b and c, it can be found that the ratio of the shorter-lifetime component to the total emission ranged from 0.57 to 0.67 in the wavelength range of 530 nm to 580 nm, whereas in the same wavelength range, the ratio of the longer-lifetime component to the total emission ranged from 0.327 to 0.428. In the wavelength range from 535 nm to 590 nm, the lifetime of the longer-lifetime component increased from 4.26 ns to 12.79 ns, and the lifetime of the shorter-lifetime component increased from 0.625 ns to 3.97 ns. Thus, from the lifetime values, we can conclude that the longer-lifetime component (exciton) is related to charged-exciton emission, whereas the shorter-lifetime (charged exciton) component includes some contribution from multi-exciton emission.

We also attempted to fit these decay traces to three-term exponentials; however, only the curves in the wavelength range of 530 nm to 570 nm could be fitted in this manner. The curves at wavelengths longer than 570 nm could only be fit to two-term exponentials. The fit parameters for the various component contributions are compared in Fig. 4d. The component with the shortest lifetime in the three-term exponential fit is attributed to multi-exciton emission, the component with the moderate lifetime is attributed to charged-exciton emission, and the component with the longest lifetime is assigned to exciton emission^8^. The ratio of the charged-exciton component to the total emission was greater than 0.5 at wavelengths from 550 nm to 590 nm, and the relative contribution of exciton emission increased from 0.13 to 0.65 with increasing wavelength from 550 nm to 610 nm. By contrast, the relative multi-exciton contribution decreased from 0.43 to 0.12 for wavelengths from 530 nm to 570 nm.

**Figure 5:**
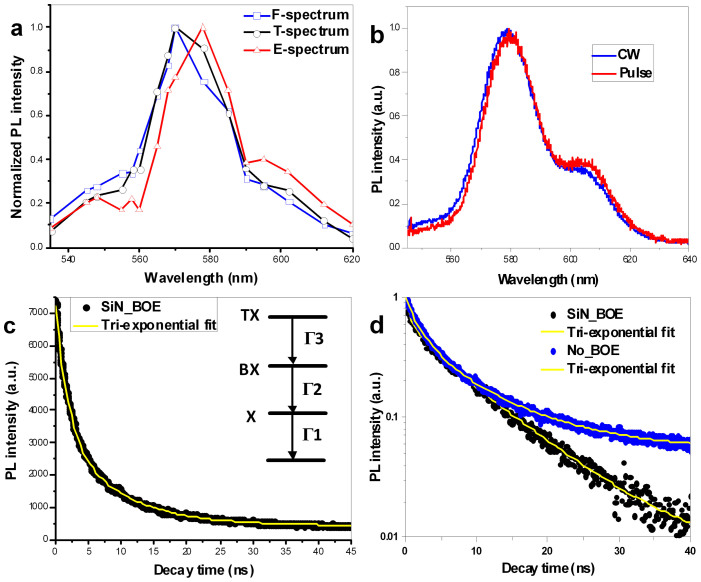
Time-resolved spectra and PL decays. (a) The PL spectra at various delay time determined from the PL decay curves at various wavelengths. (b) The PL spectrum of the CQDs determined using a spectrometer under the same conditions as in (a). (c) PL decay of CQDs on BOE-etched SiN/SiO_2_. Insert is a graphical description of exciton, bi-exciton and tri-exciton, where X represents exciton, BX bi-exciton, and TX represents tri-exciton; Γ1, Γ2, and Γ3 represent the radiative rates of exciton, bi-exciton and tri-exciton emissions. (d) Log-scaled lifetimes of emission of CQDs on unetched SiN/SiO_2_ and that on BOE-etched SiN/SiO_2_. In (c) and (d), the solid circles represent experimental results and yellow lines represent tri-exponential fits. Symbol of SiN_BOE represents the PL decay of CQDs on BOE-etched SiN/SiO_2_; Symbol of No_BOE represents the PL decay of CQDs on SiN without BOE etching.

From the decay curves at each wavelength, the PL intensity as a function of wavelength at various delay time was extracted, allowing the spectra at various delay time to be obtained. The spectra at delay time of 0 ns, 1 ns and 10 ns are compared in Fig. 5a. The spectrum acquired at a delay time of 0 ns and a gate width of 150 ps is referred to as the F-spectrum, which is predominantly associated with bi-exciton and tri-exciton emission^24^. The spectrum acquired at a delay time of 1 ns and a gate width of 150 ps is referred to as the T-spectrum and is predominantly associated with charged-exciton emission. The spectrum acquired at a delay time of 10 ns and a gate width of 150 ps is called the E-spectrum and is predominantly associated with exciton emission. Compared with the E-spectrum, the F-spectrum and T-spectrum are shifted toward bluer wavelengths (Fig. 5a). In addition, the PL intensities of the short-wavelength region at approximately 560 nm in the T-spectrum and F-spectrum are significantly stronger than that in the E-spectrum. Under excitation of pulsed laser and continue-wave (CW) laser, the spectra were measured using a spectrometer with a silicon CCD, and the spectra are presented in Fig. 5b, which may involve exciton emission and charged-exciton emission. The curve shapes shown here are similar to those of the E-spectrum and the T-spectrum presented in Fig. 5a.

The PL decay of total spectrum of CQDs on BOE-etched SiN/SiO_2_ was measured without spectrometer and fitted to three-term exponential (Fig. 5c), and the fit parameters are presented in Table 1. The ratio of the charged-exciton component to the total emission was as high as 0.469 or 0.539, approximately 2-3 times larger than the ratio obtained for the PL decay of CQDs on unetched SiN/Si. As shown in Table 1, the lifetimes, τ_x_, of the exciton emission were 10.15 ns and 14.18 ns for CQDs on BOE-etched SiN/SiO_2_ at two excitation positions. The lifetimes, τ*, of the charged-exciton emission were 2.513 ns and 4.793 ns, and the lifetimes of the multi-exciton (See insert of Fig. 5c), τ_xx_, were 0.412 ns and 1.213 ns at two positions. The PL decay of CQDs on an unetched SiN film was also measured; the results are presented in Fig. 5d and were fitted to a three-term exponential. Table 1 presents the fit parameters for two positions, which correspond to line 3 and line 4. According to these parameters, the ratio of the charged-exciton component to the total emission of CQDs on an unetched SiN was as high as 0.264, the lifetime of the charged-exciton emission was 1.876 ns, and the relative charged-exciton efficiency was 40.67%. The lifetimes observed for the BOE-etched film were longer than those observed in the unetched case. The relative charged-exciton efficiencies^23^, defined as 2τ*/τ_x_, are given in lines 1 and 2 in Table 1 for the BOE-etching cases are approximately 49.52% and 67.60% and are larger than those given in lines 3 and 4 for the unetched cases, namely, 40.67% and 41.88%. BOE etching of SiN enhances the charged-exciton efficiency, thereby suppressing Auger recombination^25^.

## Discussion

According to the results presented above, the spectral intensity of the PL from CQDs on BOE-etched SiN/SiO_2_ film decreased with increasing irradiation time. The PL spectra split into several peaks, they blue shifted with increasing irradiation time. The blue shift of the CQD spectra was similar to that observed in conventional photo-oxidation^21^, in which the spectrum contained a single emission peak. Similar behavior was also observed in our spectra of CQDs on SiN film without BOE-etching. However, for CQDs on BOE-etched SiN/SiO_2_, the spectra were found to split into several peaks. The relative contribution of the short-wavelength component increased with increasing irradiation time, whereas that of the long-wavelength component decreased. The long-wavelength component represented predominantly exciton emission, and the short-wavelength component represented predominantly charged-exciton emission. Regarding the relative contributions of the exciton and charged-exciton components, the ratio of the charged-exciton component to the total emission increased with increasing irradiation time, whereas that of the exciton component decreased. At approximately 1200 s, the relative contribution of charged exciton (the short-wavelength PL peak) exceeded that of exciton emission. According to measurements of the PL decay process, even at short irradiation time, the CQDs have already been charged. Because the emission of positively charged excitons occurs at shorter wavelengths compared with exciton emission and because of the shorter lifetime of the charged-exciton emission, the leaky modes of the SiN/SiO_2_ slab predominantly enhance the charged-exciton emission. Thus, the evolution of both peaks of the spectra with irradiation time reflects the dynamics of charging in the CQDs.

Why did the spectra split at a certain irradiation time when the SiN film had been subjected to BOE? This splitting may be related to the fact that the short-wavelength peaks of the spectra predominantly originate from the charged-exciton emission and may arise through two possible physical mechanisms. One mechanism is the Coulomb electron-electron repulsion in the BOE-etched SiN. This Coulomb interaction in the SiN will affect the electron confinement potential of the CQDs. Moreover, laser excitation at sufficiently high power intensity and with a sufficiently long duration will introduce intermixing between the CQD core and shell. This intermixing will modify the sharp electron confinement potential to a smoother one. This smoother electron confinement potential will suppress Auger recombination, causing the lifetime of the charged-exciton emission to increase. This interpretation is in consistent with the experimental results presented in Fig. 5 and Table 1. The other potential mechanism involves electron transfer. The BOE etching may bring the surface Fermi level position of the rough SiN near to the Fermi level of the CQDs, thereby facilitating electron transfer between the CQDs and the rough SiN. It was difficult to remove the CQDs from the BOE-etched SiN film using conventional washing methods, which indicated that the CQDs had conjugated to the SiN surface through covalent bonds. Moreover, the distance between the CQDs and the SiN surface is sufficiently small to give rise to resonant energy transfer. Thus, the photo-activated CQDs on the SiN form a charge-separated state, and excitons in the CQDs are positively charged. Given BOE etching with proper time, as the light irradiation time increases, the charge in the CQDs will increase, and consequently, the intensity of the charged-exciton emission component will increase. Because the spectral position of the positively charged excitons lies predominantly in the short-wavelength region of the exciton emission^6^, the charged-exciton emission is more easily enhanced by the leaky modes, which are at similar wavelengths, than is the exciton emission. Therefore, the spectral peak in the short-wavelength region originates from the strong coupling of the charged-exciton emission to the leaky modes of the SiN/SiO_2_ slab, which allows charged-exciton polariton to form. With the irradiation time increasing, the emission spectra of quantum dots shifted to blue due to photo-oxidation, as the spectra shifted to overlap with the leaky mode located at short wavelength region, the emission of CQDs was enhanced, and the coupling of the emission to the leaky mode was enhanced.

In conclusion, the PL spectra and dynamics of emission of CQDs on a SiN film were investigated by spin coating CQDs onto SiN/SiO_2_ that had been wet etched using the BOE. The PL spectra exhibited multi-peak shapes correlated with the leaky modes of the SiN/SiO_2_ slab, and the angle-resolved spectra suggested the occurrence of Rabi splitting, which indicated the formation of waveguide exciton polaritons. From comparisons of these spectra with those of CQDs on an unetched SiN film and analysis of the wavelength-dependent lifetime of the charged-exciton emission, it appeared that the wet-etched SiN film behaved as an electron acceptor. The Coulomb repulsion softened the abrupt core/shell interface and the surfaces of the CQDs, consequently decreasing the Auger recombination rate and increasing the lifetime and PL efficiency of the charged-exciton emission. The leaky modes of the SiN slab significantly enhanced the charged-exciton emission, which strongly coupled to the waveguide leaky mode to form waveguide charged-exciton polaritons.

## Methods

A silicon nitride (SiN) film with a thickness of 420 nm was grown via plasma-enhanced –chemical vapor deposition (PECVD) on top of silicon dioxide that had been thermally grown on a silicon substrate. The refractive index of the SiN was approximately 2.0. The SiN/SiO_2_ film was wet etched using BOE (HF:NH_4_F:H_2_O = 1:5:5) less than 30 s. Approximately 10 μl of a CQD water solution with a concentration of 1.6 × 10^−6^ M was drop cast onto the surface of the SiN film. The final SiN/SiO_2_ film had a thickness of 420 nm/1 μm and was coated with CdSe/ZnS CQDs, which emitted light centered at a wavelength of 585 nm under excitation by a 405 nm continuous-wave laser diode using a 40×/0.6 objective. The average excitation power was 2 mW. The emission spectra at various irradiation times were collected automatically through the same objective by a spectrometer with a Si CCD at intervals of one frame per second.

In the PL decay experiments, a 400-nm laser diode with a pulse width of 65 ps was used as the pump light. A 40×/0.60 objective was used as the focusing lens and as the collection lens to filter the emission from the CQDs. The collected signal was then sent to a monochromator (Princeton Instrument, SP-2500) with a photomultiplier tube (Becker & Hickl-bh, PMC-100-20) and then to a time correlated single photon counting (TCSPC, Becker & Hickl-bh, SPC-150) system for lifetime analysis.

## Author Contributions

X.S.X. designed research, performed mostly experiment, X.Y.L. performed part of experiment, and X.S.X. wrote the main manuscript text. All authors reviewed the manuscript.

## Additional Information

**How to cite this article**: Xu, X. & Li, X. Enhanced emission of charged exciton-polaritons from colloidal quantum dots on an SiN/SiO2 slab waveguide. *Sci. Rep.* **5**, 9760; doi: 10.1038/srep09760 (2015).

## Supplementary Material

Supplementary information Supplementary information

## Figures and Tables

**Table 1:** The three-term exponential fit parameters for PL decay. SiN_BOE represents the PL decay of CQDs on BOE-etched SiN/SiO_2_; No_BOE represents the PL decay of CQDs on SiN without BOE etching. The symbols τ_x_, τ*, τ_xx_, A_x_, A*, A_xx_, QY_xx_, and QY* represent the exciton lifetime, the charged-exciton lifetime, the lifetime of multi-exciton emission, the ratios of exciton, charged-exciton and multi-exciton emissions to total emission, the efficiency of multi-exciton emission, and the relative efficiency of charged-exciton emission, respectively

SiN_CQD	τ_x_ (ns)	τ* (ns)	τ_xx_ (ns)	A_x_ ratio	A* ratio	A_xx_ ratio	τ*/τ_xx_	QY_xx_ (%,4*τ*_xx_/*τ*_x_,%)	QY* ( = 2τ*/τx,%)
BOE, P1	10.150	2.513	0.412	0.350	0.539	0.110	6.099	16.24	49.52
BOE, P2	14.180	4.793	1.213	0.330	0.469	0.202	3.950	34.12	67.60
No-BOE	9.225	1.876	0.287	0.499	0.264	0.253	6.536	12.44	40.67
No-BOE	8.954	1.875	0.290	0.493	0.184	0.322	6.465	12.96	41.88

## References

[b1] Efros A. L. & Rosen M. Random telegraph signal in the photoluminescence intensity of a single quantum dot. Phys. Rev. Lett. 78, 1110 (1997).

[b2] Krauss T. D. & Brus L. E. Charge, polarizability, and photoionization of single semiconductor nanocrystals. Phys. Rev. Lett. 83, 4840 (1999).

[b3] Frantsuzov P., Kuno M., Janko B. & Marcus R. A. Universal emission intermittency in quantum dots, nanorods and nanowires. Nat. Phys. 4, 519–522 (2008).

[b4] Chepic D. *et al.* Auger ionization of semiconductor quantum drops in a glass matrix. J. Lumin. 47, 113–127 (1990).

[b5] Efros A. L. Nanocrystals: Almost always bright. Nat. Mater. 7, 612–613 (2008). 18654583 10.1038/nmat2239

[b6] Saad A., Bakr M., Azzouz I. & Abou Kana M. T. Effect of temperature and pumping power on the photoluminescence properties of type-II CdTe/CdSe core-shell QDs. Appl. Surf. Sci. 257, 8634–8639 (2011).

[b7] Wang X. Y. *et al.* Non-blinking semiconductor nanocrystals. Nature 459, 686–689 (2009). 19430463 10.1038/nature08072

[b8] Javaux C. *et al.* Thermal activation of non-radiative Auger recombination in charged colloidal nanocrystals. Nat. Nanotechnol. 8, 206–212 (2013). 23396313 10.1038/nnano.2012.260

[b9] Robel I., Subramanian V., Kuno M. & Kamat P. V. Quantum dot solar cells. Harvesting light energy with CdSe nanocrystals molecularly linked to mesoscopic TiO2 films. J. Am. Chem. Soc. 128, 2385–2393 (2006). 16478194 10.1021/ja056494n

[b10] Jin S., Song N. & Lian T. Suppressed blinking dynamics of single QDs on ITO. Acs Nano 4, 1545–1552 (2010). 20170100 10.1021/nn901808f

[b11] Huang J., Stockwell D., Huang Z., Mohler D. L. & Lian T. Photoinduced ultrafast electron transfer from CdSe quantum dots to re-bipyridyl complexes. J. Am. Chem. Soc. 130, 5632–5633 (2008). 18393497 10.1021/ja8003683

[b12] Song N., Zhu H., Jin S., Zhan W. & Lian T. Poisson-distributed electron-transfer dynamics from single quantum dots to C60 molecules. ACS Nano 5, 613–621 (2010). 21190376 10.1021/nn1028828

[b13] Greenham N. C., Peng X. & Alivisatos A. P. Charge separation and transport in conjugated-polymer/semiconductor-nanocrystal composites studied by photoluminescence quenching and photoconductivity. Phys. Rev. B 54, 17628–17637 (1996). 10.1103/physrevb.54.17628 9985889

[b14] Sharma S. N., Pillai Z. S. & Kamat P. V. Photoinduced charge transfer between CdSe quantum dots and p-phenylenediamine. J. Phys. Chem. B 107, 10088–10093 (2003).

[b15] Hamada M. *et al.* Single-molecule photochemical reactions of Auger-ionized quantum dots. Nano Rev. 2, 6366 (2011). 10.3402/nano.v2i0.6366 PMC3226428 22132300

[b16] McGuire J. A. *et al.* Spectroscopic signatures of photocharging due to hot-carrier transfer in solutions of semiconductor nanocrystals under low-intensity ultraviolet excitation. ACS nano 4, 6087–6097 (2010). 20939512 10.1021/nn1016296

[b17] Ellenbogen T. & Crozier K. B. Exciton-polariton emission from organic semiconductor optical waveguides. Phys. Rev. B 84, 161304 (2011).

[b18] Deng H., Weihs G., Santori C., Bloch J. & Yamamoto Y. Condensation of semiconductor microcavity exciton polaritons. Science (New York, N.Y.) 298, 199–202 (2002). 10.1126/science.1074464 12364801

[b19] Tassone F. & Yamamoto Y. Exciton-exciton scattering dynamics in a semiconductor microcavity and stimulated scattering into polaritons. Phys. Rev. B 59, 10830 (1999).

[b20] Amo A. *et al.* Exciton–polariton spin switches. Nat. Photon 4, 361–366 (2010).

[b21] Wang X. Y., Zhang J. Y., Nazzal A. & Xiao M. Photo-oxidation-enhanced coupling in densely packed CdSe quantum-dot films, *Appl*. Phys. Lett. 83, 162–164 (2003).

[b22] Xu X. S. Enhanced trion emission from colloidal quantum dots with photonic crystals by two-photon excitation. Sci. Rep. 3, 3228 (2013). 24231669 10.1038/srep03228 PMC3828571

[b23] Lin H. *et al.* Strong coupling of different cavity modes in photonic molecules formed by two adjacent microdisk microcavities. Opt. Exp. 18, 23948–23956 (2010). 10.1364/OE.18.023948 21164741

[b24] Fisher B. *et al.* Room-Temperature Ordered Photon Emission from Multiexciton States in Single CdSe Core-Shell Nanocrystals. Phys. Re. Lett. 94, 087403 (2005). 10.1103/PhysRevLett.94.087403 15783930

[b25] Bae W. K. *et al.* Controlling the influence of Auger recombination on the performance of quantum-dot light-emitting diodes. Nat. Commu. 4, 2661 (2013). 10.1038/ncomms3661 PMC3826634 24157692

