# Supplementary material for: Enhanced emission of charged-exciton polaritons from colloidal quantum dots on a SiN/SiO2 slab waveguide
Source: Sci Rep. 2015 May 19;5:9760. doi: 10.1038/srep09760 (PMC4437026; doi:10.1038/srep09760)
Supplement: Supplementary information [file srep09760-s1.doc]

***Supplementary information***

**Enhanced emission of charged exciton-polaritons from colloidal quantum dots on a SiN/SiO_2_ slab waveguide**

**Xingsheng Xu*, Xinyun Li**

State Key Laboratory of Integration Optoelectronics, Institute of Semiconductors, Chinese Academy of Sciences, Beijing 100083, China

* Email: xsxu@semi.ac.cn

The spectra and their tri-Gaussian fits are present in Fig. S1(a) and (b). The spectrum acquired after a short irradiation time of 2 s is present in Fig. S1(a), and the spectrum acquired after an irradiation time of 1000 s is present in Fig. S1(b). The spectrum acquired after irradiation time of 100 s are fitted to a tri-Gaussian function and a bi-Gaussian function, the corresponding fitted curves are present in Fig. S1(c) and (d), respectively. The spectra at different irradiation time are compared in Fig. S1 (e). Two dotted lines connect the wavelength positions of short-wavelength peak and long-wavelength peak, respectively. The spectra are split and an anticrossing between the two spectral peaks (two modes) is displayed. The spectra of CQDs on unetched SiN were found to exhibit very few multi-peak characteristics. The spectrum of CQDs on unetched SiN is shown in Fig. S1(f). This spectrum contains only one peak, which can be fitted to Gaussian function.

**Figure S1 The spectrum of CQDs on SiN/SiO_2_ with a thickness of 420 nm and corresponding fitting.** (a) BOE etching time of 100 s and bi-Gaussian fit, (b) BOE etching time of 100 s and tri-Gaussian fit, (c) BOE etching time of 2 s and bi-Gaussian fit, (d) BOE etching time of 1000 s and bi-Gaussian fit, (e) Comparison of the spectra at different irradiation time. (f) The spectrum of CQDs on SiN/SiO_2_ without BOE etching and its Gaussian fit.

The spectra under different irradiation time fitted into bi-Gaussian function are present in Fig. S2, the yellow lines are the fitting curves, and the fitted two Gaussian terms are also shown as dotted-red line and dotted-blue line in corresponding branch figures. The spectrum with irradiation time 8 s is shown in Fig. S2(a), the spectrum is mainly centered at 585.4 nm, and there are two weak spectral peaks in both long-wavelength side and short-wavelength side. With irradiation time increasing to 200 s (Fig. S2(b)), the total PL intensity increases slightly due to photo-oxidation, the centre wavelength shifts to 581 nm, and the PL intensity of side peak at short wavelength increased a little compared with that in Fig. S2(a). With irradiation time increases more to 250 s, it can be found that the main peak blue shifts to 580.5 nm, and the side peak at short-wavelength increased to more than half intensity of the main peak at 580.5 nm, while the side peak at long-wavelength almost disappeared (Fig. S2(c)). As irradiation time increases to 500 s in Fig. S2(d), it can be found that the side peak at short wavelength 553.7 nm increases to similar intensity to that of the main peak at 577.7 nm. The spectrum with irradiation time 570 s is shown in Fig. S2(e), where the side peak at short wavelength is higher than that at 581 nm.

**Figure S2 The PL spectra of CQDs on a BOE-etched SiN/SiO_2_ film for various irradiation time:** (a) 8 s, (b) 200s, (c) 250 s, (d) 500 s. (e) 570 s. (a)-(e) The yellow curved lines are represent bi-Gaussian fits, the dotted-curves are the Gaussian fits.

**Figure S3 The bi-Gaussian fitted parameters of the spectra presented in Fig. S2**(a) The center wavelengths as function of light irradiation time. (b) Spectral widths as function of light irradiation time. (c) Ratios of the short-wavelength component and the long-wavelength component to the total emission. (d) Enlarged the center wavelengths as function of light irradiation time. C_l_, C_s_, are the center wavelengths of the long-wavelength component and the short-wavelength component, respectively; W_l_, W_s_ are the spectral widths of the long-wavelength component and the short-wavelength component, respectively; R_l_, R_s_ are the ratios of the long-wavelength component and the short-wavelength component to the total emission, respectively; E_l_, E_s_ are the energy of the long-wavelength component and the short-wavelength component, respectively. (e) The data presented in Fig. 2(a) in units of meV instead of nm.

The spectra under different irradiation time are fitted into bi-Gaussian function; the yellow lines are the fitting curves. The fitted parameters are displayed in Fig. S3. During 68 min, the center wavelength of the short-wavelength component in initial time is around 555 nm, the initial center wavelength of the long-wavelength component around 580 nm, both of them shift to blue slowly with irradiation time (Fig. S3(a)). Seen from Fig. S3(b), the spectral width of the long-wavelength component varies from 15.3 to 13.9 nm, while the spectral width of the short-wavelength component dramatically increases from 7.7 to 14.9 nm. The ratio of the long-wavelength component to the total component decreases from 0.88 to 0.47, while the ratio of the short-wavelength component to the total component increases from 0.12 to 0.53 in 68 min (Fig. S3(c)). Correspondingly, the amplitude of the long-wavelength component decreases dramatically whereas the real amplitude of the short-wavelength component increases slowly, and the total amplitude (representing the PL intensity) also dramatically drops in the irradiation time 68 min. The modes as function of the irradiation time is shown in Fig. S3(d). At initial irradiation time of 1 s, the interval between the long-wavelength and the short-wavelength mode is 27 nm. As the irradiation time increases to 22 min, the deviation decreases to 24.1 nm. With irradiation time increases to 68 min, the deviation increases to 25.6 nm. Therefore, two modes of short-wavelength and long-wavelength strongly couples to each other, as well as exciton and the charged exciton inter acts with each other.

**Figure S4**. **PL decay at various wavelengths and the fit parameters for the PL decay fitted to multi-exponential functions (The same point to that of Fig. 4).** PL decay at various wavelengths: (a) 555 nm, (b) 585 nm. The solid black lines represent experimental results, and the green lines are fitted curves obtained using two-term exponential function.

**Figure S5 PL decay at various wavelengths (Another point to that of Fig. 4)**: (a) 610 nm, (b) 600 nm, (c) 590 nm, (d) 578 nm, (e) 570 nm, (f) 560 nm, (g) 550 nm, (h) 540 nm. The black hollow-circle lines represent experimental results, and the green lines are fitted curves obtained using two-term exponential function.

**Figure S6 Fit parameters for the PL decay at various wavelengths fitted to tri-exponential functions (The same point to that of Fig. 4).** (a) The lifetimes for exciton emission, (b) The lifetimes for charged-exciton emission, (c) The lifetimes for multi-exciton emission, (d) The component ratios from the tri-exponential fitting of PL decay at various wavelengths. (e) The PL spectra at various delay time determined from the PL decay curves at various wavelengths.

As PL decays at different wavelength from CQDs in another position of SiN/SiO2 are displayed in Fig. S5. The PL decays are fitted to three-termed exponentials and the fitted parameters are collected in Fig. S6. As wavelength changing in the range of 535 nm to 610 nm, the lifetimes for the long-lifetime term are in the range of 9.11 ns and 24.08 ns, the lifetime for the middle lifetime term are in the range 2.08 ns and 7 ns, this term is attributed to exciton emission; this term is attributed to charged-exciton emission; while the lifetime for the short-lifetime term is in the range of 0.15 ns and 1.74 ns, this terms is attributed to multi-exciton emission. In the wavelength ranging from 535 nm and 580 nm, the corresponding ratios of the charged-exciton term are in the range 0.47 and 0.62. The ratios of the multi-exciton components to the total component are 0.12 and 0.40, while the ratios of the exciton component are 0.16 and 0.39. Moreover, the curves of all the parameters including, lifetimes and component ratios as function of the wavelength, fluctuated with the wavelength.
